# Proof of Concept of Biopolymer Based Hydrogels as Biomimetic Oviposition Substrate to Develop Tiger Mosquitoes (*Aedes albopictus*) Cost-Effective Lure and Kill Ovitraps

**DOI:** 10.3390/bioengineering9070267

**Published:** 2022-06-21

**Authors:** Marco Friuli, Claudia Cafarchia, Andrea Cataldo, Riccardo Paolo Lia, Domenico Otranto, Marco Pombi, Christian Demitri

**Affiliations:** 1 Department of Engineering for Innovation, University of Salento, 73100 Lecce, Italy; andrea.cataldo@unisalento.it (A.C.); christian.demitri@unisalento.it (C.D.); 2 Department of Veterinary Medicine, University of Bari, Valenzano, 70010 Bari, Italy; claudia.cafarchia@uniba.it (C.C.); riccardopaolo.lia@uniba.it (R.P.L.); domenico.otranto@uniba.it (D.O.); 3 Dipartimento di Sanità Pubblica e Malattie Infettive, Università di Roma “Sapienza”, 00185 Rome, Italy; marco.pombi@uniroma1.it

**Keywords:** biopolymers, biomimetics, hydrogels, *Aedes albopictus*

## Abstract

Pest management is looking for green and cost-effective innovative solutions to control tiger mosquitoes and other pests. By using biomimetic principles and biocompatible/biodegradable biopolymers, it could be possible to develop a new approach based on substrates that selectively attract insects by reproducing specific natural environmental conditions and then kill them by hosting and delivering a natural biopesticide or through mechanical action (biomimetic lure and kill approach, BL&K). Such an approach can be theoretically specialized against tiger mosquitoes (BL&K-TM) by designing hydrogels to imitate the natural oviposition site’s conditions to employ them inside a lure and kill ovitraps as a biomimetic oviposition substrate. In this work, the hydrogels have been prepared to prove the concept. The study compares lab/on-field oviposition between standard substrates (absorbing paper/masonite) and a physical and chemically crosslinked hydrogel composition panel. Then the best performing is characterized to evaluate a correlation between the hydrogel’s properties and oviposition. Tests identify a 2-Hydroxyethylcellulose (HEC)-based physical hydrogel preparation as five times more attractive than the control in a lab oviposition assay. When employed on the field in a low-cost cardboard trap, the same substrate is seven times more capturing than a standard masonite ovitrap, with a duration four times longer.

## 1. Introduction

Pest management is crucial both for human health and crop protection [1]. During the last years, it faced new challenges such as resistance to insecticides and new regulations about chemical use. Among the numerous solutions proposed, it coped by developing strategies to reduce the volume of pesticides employed (improving the delivery and targeting) or shifting to less dangerous insecticides. Among the most employed natural biopesticides, there are microbial larvicides (e.g., *Bacillus thuringiensis var. Israelensis* or *Wolbachia)* [2] natural essential oils (e.g., oil of pennyroyal (*Mentha pulegium*) or *Ruta chalepensis* [3,4,5] entomopathogenic fungi, such as *Metarhizium anisopliae* or *Beauveria bassiana* (Bb) [6,7,8]. Precision pest management approaches, PPM [9,10], are diametrically opposite to random chemical spraying. They aimed to reduce the volume of pesticides and side effects through more targeted delivery [11,12]. Lure and kill (L&K) has been among the most successful PPM approaches. It uses semiochemical stimuli, e.g., sexual/feeding pheromones, as attractants to trigger insects to interact with poisoned products (e.g., baits, traps, or systems for insecticide controlled release [13]) rather than chase them by spraying [14,15,16,17]. However, lure and kill success is conditioned on the functioning of the attractive systems. Some classes of insects are less/not responsive to semiochemical stimuli or do not have a specific attractant. For example, mosquitoes [18], particularly tiger mosquitoes (*Aedes albopictus*, a significant disease vector), are not responsive to standard attractants [19,20,21,22,23,24].

Natural bioinsecticides have a low environmental impact but present also low efficacy in field conditions and at a higher cost than chemical pesticides [25]. They are mainly delivered by spraying and are scarcely employable in PPM approaches because commercially available L&K devices are generally not optimized to support their survival and infection mechanisms effectively (e.g., non-biocompatible substrates, fast desiccation, lack of nutrients, etc.) [26,27]. Consequently, new attractive systems have to be developed and, eventually, designed for bioinsecticide delivery to match PPM practices.

For this purpose, in a previous work [28], we conceptualized an innovative PPM approach called biomimetic lure and kill (BL&K), which is based on biocompatible biopolymers and biomimetics principles already widely employed in the medical field [29,30,31,32]. Within this context, we can define biocompatible as a material that interacts without toxic effects on the hosted biological system and is able to promote and sustain its growth. Well-known examples of biocompatible biopolymers are polysaccharides such as cellulose and its derivatives, and alginates [33,34]. In BL&K, we claimed that a biocompatible biomaterial could be exploited to host a natural bioinsecticide [4,35,36,37,38,39,40] and, at the same time, to attract the insects by designing it to mimic the conditions of the natural environment associated with a particular insect’s behavior (breeding, taking shelter, resting, etc.) (biomimetic lure) [41].

Consequently, it could have a lure and kill action without chemical pesticide and semiochemical use.

We proposed biomaterial-based hydrogels as a potential platform to realize BL&K. They are a class of material mainly made of water that can be formulated and processed to adapt their physical and chemical parameters (such as water content, rheological and mechanical properties, etc.) to replicate several environmental conditions, as required for biomimetic lure [42,43,44,45,46]. Furthermore, biomaterial-based hydrogels are generally biocompatible, as required to host a natural bioinsecticide.

For example, biomaterial-based hydrogels might be employed to specialize in the BL&K approach to control tiger mosquitoes (BL&K-TM) in order to obtain a lure and kill oviposition substrate for lethal ovitraps. For that purpose, they can be designed to contain a bioinsecticide and mimicry that features that drive gravid tiger mosquito females during the selection of the oviposition substrate/sites in the natural environment [47]. The entomopathogenic fungus *Beauveria bassiana* (Bb) [48] could be one of the possible bioinsecticides that can be hosted by the hydrogel. Bb is safe for human and animal health, but it infects different arthropods and mosquito species [8,49], killing them within a period long enough to allow the autodissemination process [50].

Ovitraps are widely spread as a monitoring tool but underemployed as a lure and kill device for tiger mosquitoes. We claimed that the hydrogel-based oviposition substrate could be more effective than a simple and not specially designed substrate. Furthermore, we supposed that this approach could promote the choice of ovitraps and bioinsecticides as a mass control method [50,51,52,53,54,55,56]. Not just by providing a more attractive oviposition substrate but else too by reducing ovitraps’ costs for maintenance by extending their working period (e.g., through hydrogel’s water retention ability), avoiding the disposal (through biodegradation), and by supporting the activity of the bioinsecticide to improve its efficacy in field conditions. This way, a standard low-cost monitoring ovitrap can be converted into a green, cost-effective, and selective L&K control device.

The reduced efficacy and high ecological impact of traditional pest management methods highlight the need to research innovative, effective, and sustainable control methods. The Biomimetic Lure and Kill approach proposed herein, modulated by TE and based on the principles of biomimicry, employs the potential for applicability to several pest species and is already technically feasible, as all the technologies necessary to design and configure materials with specific characteristics already exist. This approach might be cost-competitive, increasing the effectiveness of substrates and devices as much as possible. The role of material engineering is instrumental in finding the best materials and fitting processing, technically and economically, in a continuous transfer of knowledge from materials engineering to entomology. Developing a new pest control approach requires a multidisciplinary process, and strong interaction among different research areas is needed, in particular, to find specific behavioral patterns in insects and quantify their driver parameters. This study aims to validate the tiger mosquito dedicated version of the approach (BL&K-TM) by proving oviposition on hydrogels and, at the same time, finding a formulation able to maximize the ovitrap’s performance in terms of luring effect and active period. Even though it can be considered a preliminary study and further studies are necessary to verify the possibility of hosting a bioinsecticide and to evaluate the lethality of the system, it can mark a path to developing new ideas and approaches for the targeted control of other classes of pests.

## 2. Materials and Methods

### 2.1. Rationale for Oviposition Parameters Selection

Tiger mosquitoes lay their eggs on the wet inner walls of natural/artificial water containers waiting for the water level to rise to submerge them causing hatch [57]. The primary natural environments exploited as oviposition sites are hollow trunks, plant leaves, and muddy embankments. Numerous factors could influence the search, detection, identification, and selection of a specific oviposition substrate. However, pH, salinity, turbidity, repellent effect of some chemical compounds (such as pesticides) [58,59,60,61,62,63,64,65], substrate texture, morphology, slope, moisture content, and water release rate have been frequently indicated as determinants in the final choice of a site [66,67,68], but they are not identified and quantified univocally into limited ranges [69,70]. A panel of possible qualitative ranges is resumed in Table 1. It has been employed as a starting point for selecting polymer type and hydrogel features to be tested during oviposition assays.

### 2.2. Rationale in Biopolymer and Substrate Composition Selection

The 2-Hydroxyethylcellulose (HEC) and sodium alginate (SA) were chosen due to their hydrophilic nature and their chemical composition similar to natural substrates. Solutions of HEC and SA were produced in a wide range of concentrations by weight (wt%) and processed to form physical (non-crosslinked) and chemically crosslinked (CL) hydrogels to obtain different substrate textures ranging from ‘liquid’ to ‘rigid’.

### 2.3. Materials

2-Hydroxyethylcellulose powder (HEC, Sigma-Aldrich, St. Louis, MI, USA, average Mw 720000) and sodium alginate powder (SA, SAFC, Sigma-Aldrich, St. Louis, MI, USA, average Mw 120000) were the biopolymers used to produce hydrogels. HEC-based chemically crosslinked hydrogels have been synthesized using Poly-(ethylene glycol)-diglycidyl-ether (PEGDE, Sigma-Aldrich, St. Louis, MI, USA, average Mw 500, liquid) as crosslinker and NaOH, (Sodium hydroxide BioXtra, ≥98%, Sigma-Aldrich, St. Louis, MI, USA, pellets (anhydrous)) as catalyst, then neutralized with HCl (HCl 37%, Sigma-Aldrich, St. Louis, MI, USA). SA-based chemically crosslinked hydrogels have been crosslinked using calcium chloride (CaCl_2_, Anhydro Beads, ≥99.9%, Sigma-Aldrich, St. Louis, MI, USA). Sorbitol (S, D-sorbitol 99%, Sigma-Aldrich, St. Louis, MI, USA), NaCl (Sodium chloride BioXtra, ≥99.5%, Sigma-Aldrich, St. Louis, MI, USA), NaOH, (Sodium hydroxide BioXtra, ≥98%, (anhydrous), Sigma-Aldrich, St. Louis, MI, USA), Lactic acid (L-(+)-Lactic acid ≥98%, Sigma-Aldrich, St. Louis, MI, USA), and lab-made masonite maceration were added to preparations respectively to change water evaporation rates and generate ranges of salinity, pH, and turbidity. The masonite maceration was lab-produced by boiling 3 new masonite sticks (3 × 15 cm) for 10 min in 0.5 L of distilled water. Sticks were then soaked in water for 10 days to obtain a dark brown liquid to be diluted with distilled water to the desired color. Standard white absorbing paper and masonite sticks were used in oviposition assays.

### 2.4. Physical Hydrogels Preparation

Physical hydrogels (not crosslinked) were produced with the same protocol for HEC and SA. HEC and SA powders 2–30 wt% (HEC2-30 and SA2-30) to distilled water and stirred at 1500–2000 rpm at room temperature for about 30 min.

### 2.5. Crosslinked (CL) Hydrogels Preparation

#### 2.5.1. Hydroxyethylcellulose Crosslinked Hydrogels, CL-HEC

HEC Crosslinked hydrogels (2–30 wt%, CL-HEC2-30) were produced through the protocol already reported in Kono et al. [71]. HEC powder was added to distilled water (90 mL) containing PEGDE (4 mL) and stirred at 1500–2000 rpm at room temperature for about 30 min (solution A). Separately, a solution (solution B) of distilled water (10 mL) and NaOH (4 g) was prepared. After complete mixing, solution B was added to solution A and incubated at room temperature overnight. A third solution (neutralization solution, solution C) of distilled water (96 mL) and HCl (4.10 mL) was prepared to neutralize NaOH. Hydrogels were the following: (i) cut into pieces and immersed in solution C until neutral pH was restored (ii) repeatedly washed in distilled water, (iii) submerged until swollen state (or equilibrium state).

#### 2.5.2. Sodium Alginate Crosslinked Hydrogel CL-SA2-30

SA crosslinked hydrogel samples (2–30 wt%, CL-SA2-30) were prepared by adding SA powder to distilled water (e.g., 2 g for CL-SA2) and stirred at 1500–2000 rpm at room temperature for about 30 min. In another container a solution of calcium chloride (1 Molar) was prepared and used as crosslinking agent for SA [72]. Due to the difficulties in shaping CL-SA to perform oviposition test, a masonite stick (2 × 10 cm), surely not repellent oviposition substrate, were dip-coated into SA solution to obtain a 2–3 mm layer (5 cm length). Then, the coated stick was immersed in CaCl_2_ solution at 35 °C for 1 h to promote the crosslinker diffusion.

### 2.6. Preparation of Different Formulations of the Best Performing Hydrogel

Different formulations of the best performing hydrogel (identified through the oviposition assay 1, see Section 2.7.3) were prepared to evaluate the effect of other parameters on oviposition preference.

#### 2.6.1. Hydrogels with Different Salinity

Samples having different salinities values were obtained by adding NaCl to the initial polymer-water solution while controlling salinity value with a portable conductivity meter (Bormac model 5 COND 70+). The solutions were adjusted to obtain a salinity range of 0–5%.

#### 2.6.2. Hydrogels with Different pH

Samples having different pH values were obtained by adding NaOH or lactic acid to the initial polymer-water solution while controlling the pH value with a Mettler Toledo model pH8 benchtop pH-meter. The solutions were adjusted to obtain a pH range of 4.5–10 (in particular the pH ranges introduced were <4.5; 5.5–4.5; 6.5–5.5; 6.5–7.5; 8–9; 10. The control had pH = 6.5.

#### 2.6.3. Hydrogels with Sorbitol

Hydrogels with different water release rates were obtained by adding D-Sorbitol (0–10 wt%, S0–S10) to the initial polymer-water solution [73].

#### 2.6.4. Hydrogels with Different Turbidity

**Figure 1:**
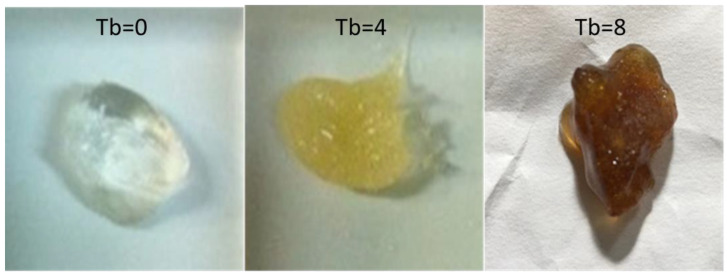
Example of different turbidity levels on hydrogel color.

Samples having different turbidities were prepared to obtain a color variation in gels (Figure 1). They were obtained by adding the initial polymer-water solution to the lab-prepared masonite maceration (obtained as previously described) while controlling the turbidity with a densitometer (Biosan DEN 1). The solutions were adjusted to obtain a turbidity range of 0–10 McFarland (Tb0-Tb10).

### 2.7. Oviposition Assays on Ades albopictus

#### 2.7.1. *Aedes albopictus* Colony

An *Aedes albopictus* colony was raised in an insectary placed at the Parasitology Section of the Department of Public Health and Infectious Diseases of the Sapienza University of Rome. Mosquitoes have been in captivity for 50 generations before being used in the assay. Colony breeding conditions were the following: T = 25 ± 1 °C, RH 80%, light hours/darkness hours ratio was 14:10. Mosquitoes made a blood meal 48 h before the oviposition test [74] and have been fed using a sugar-water solution during the oviposition assays.

#### 2.7.2. Lab Oviposition Assay Design

At the end of the feeding, 30 gravid and fully engorged females for each tested substrate were manually selected from the colony and maintained in a cage containing the tested substrates, and the control placed inside a white plastic container filled with 30 mL of distilled water. Oviposition assays were performed by comparing inside the same cage samples having the same features (e.g., HEC16 vs. SA16 or CL-HEC16 vs. CL-SA16) against absorbing paper as control. In total, 10 g of physical hydrogels were spread on 2 × 5 cm cardboard and then placed into the plastic container, whereas 2 × 5 cm CL samples were leaned on the cardboard. All the tests were performed in triplicate in a controlled environment at T 25 ± 1 °C, RH 80%. On a timestep basis, samples were removed from cages, and eggs were counted using an optical microscope. Oviposition results are expressed for each sample through the ratio T/C ± Standard deviation (SD), where T = total number of eggs laid on the tested sample; C = total number of eggs laid on the control. A sample reached more eggs than the control when T/C > 1.

#### 2.7.3. Oviposition Assay 1: Proof of Concept, Effects of Hydrogel Type and Polymer Concentration

HEC2-30, SA2-30, CL-HEC2-30, and CL-SA2-30 have been tested for 24 h as oviposition substrates using absorbing paper as control and following the protocol described above. This scouting oviposition test was conducted to evaluate the feasibility of oviposition on the chosen hydrogels and find the best performing substrate composition.

#### 2.7.4. Oviposition Assay 2: Effects of Salinity, pH, Sorbitol and Turbidity

The best performing substrate in oviposition assay 1 was prepared as previously described by varying its pH (from 4.5 to 10), salinity (0–5%), sorbitol concentration (0–10 wt%), and turbidity (0–10 McF). The different compositions were separately tested as oviposition substrate in lab oviposition assay 2 for 24 h using absorbing paper as control and following the protocol described above.

#### 2.7.5. Oviposition Assay 3: Comparison between Best Performing Formulation over 2 Weeks

The best performing hydrogel compositions in oviposition assays 1 and 2 were compared in a third two-week-long oviposition assay, performed by using the same protocol previously described. They were all proposed in the same cage against a black standard oviposition trap with masonite sticks in distilled water and absorbing paper as control. The initial gravid female mosquitoes were replaced after the first week with other gravid females prepared as described above. During the experiment, mosquitoes were fed using a sugar-water solution that was replaced at the end of the first week.

#### 2.7.6. On-Field Oviposition Assay on Best Performing Hydrogel Formulation over 30 Days

A hydrogel having all the most attractive features found in the lab oviposition assay 3 was employed in a single preliminary test in field conditions. About 200 g of hydrogel was spread and used as oviposition substrate inside a commercial low-cost cardboard trap (cockroaches-like trap) having an active surface of 12 × 15 cm. The hydrogel was compared to a masonite black standard oviposition trap filled with 200 mL of distilled water and provided with a 2 × 15 cm masonite stick as oviposition substrate. Three traps for each substrate were placed for 30 days in a restricted area (15 × 15 m backyard) in Settimo Milanese (Milan, Italy) throughout September 2019. Average environmental temperature and humidity were 23 ± 1 °C and 75% RH during the test period (data from Fondazione Osservatorio Metereologico Milano Duomo). The number and type of eggs were evaluated every 15 days through microscopical analysis (without removing them from the substrate). Furthermore, the traps’ weights were monitored daily to evaluate water evaporation from the substrates.

### 2.8. Oviposition Substrates Characterization

Characterization was performed downstream oviposition. Physical hydrogels have been characterized since promising as oviposition substrates to give a possible explanation for substrate preferences in oviposition and to evaluate their use inside ovitraps. Characterization of crosslinked hydrogels has been made to explain their repellent effect.

#### 2.8.1. Calorimetric Analysis of Free Water Content

Calorimetric analysis was performed on HEC2-30 and SA2-30 and standard controls, i.e., absorbing paper and masonite (soaked for 4 days in distilled water) to evaluate the free water percentage (FW%). The analysis was performed through differential scanning calorimetry (DSC) using a Q2000 Series DSC from TA Instruments DSC. Samples were subjected to a thermal cycle in a nitrogen atmosphere composed of a cooling ramp of 5 °C/min to −20 °C and then a heating ramp of 5 °C/min up to 50 °C [75]. Then, melting peak areas were measured with the specific functionality of the software Q2000 Series software analysis. FW% values have been reported for each sample.

#### 2.8.2. Effect of Sorbitol and Polymer Concentration on Water Release at Controlled T and RH

Weight loss assay was performed on HEC16/S0-10 and HEC16, HEC8, and HEC2 with and without 6 wt% of sorbitol, to evaluate the effect of the humectant and polymer concentration on water release. Hydrogel samples were placed singularly in a plastic container and kept in a Binder climatic chamber at 25 °C, RH = 80% (field-like conditions). Samples were weighed every 24 h, and results have been reported as values normalized to the initial weight.

#### 2.8.3. Water Release in Field-like Conditions at Controlled T and RH

Weight loss assay was performed on HEC16 and HEC16/S6 hydrogel formulations and masonite in a usual trap setup to evaluate gel behavior in a field-like setup and conditions. About 200 g of each hydrogel was spread on a silicone paper stripe bonded to the inner wall of a plastic commercial trap (Polytrap©, Gea Srl, Milan, Italy). A masonite trap was employed as control by placing a 2 × 15 cm masonite stick in the same type of trap under the same conditions immersed in 200 mL of water. The samples (trap + hydrogel) and the control trap were kept in a Binder model KBF 115 climatic chamber at 25 °C and 80% RH (field-like conditions) for 24 days. Samples and controls were weighed daily, and results have been reported as values normalized to the initial weight. At the end of the testing period, HEC16 and HEC16/S6 and masonite were recovered from the traps and tested through DSC analysis performed as described above to evaluate the residual free water.

#### 2.8.4. Gel Viscosity Measurement

Shear viscosity was measured on HEC2-30 and SA2-30 hydrogels with a single shear rate test with a Malvern Kinexus Pro rotational rheometer in a plate-plate configuration (50 s^−1^ of shear rate for 5 min, 25 °C, and atmospheric pressure). Viscosity was investigated as related to substrate texture and water evaporation rate [76,77]. Shear viscosity was also evaluated in sorbitol and masonite maceration prepared samples, but no influence on rheological behavior was found. The results were not reported.

#### 2.8.5. Yield Stress Measurement

Yield stress τ_0gel_ was measured on HEC2-30 and SA2-30 to evaluate the lowest polymer concentration spreadable on a vertical surface (such as the trap’s inner walls) without flowing down under the shear stress τ due to its weight, F_w_. The no-flow condition is τ_0ge_ > τ.

τ_0gel_ was evaluated with a Malvern Kinexus Pro rotational rheometer (rSpace software 2.0, Netzsch, Verona, Italy) using a standard test (based on Bingham model) in a shear rate range of 0–100 s^−1^ in the plate-plate configuration at 25 °C. τ was calculated as F_w_/A_t_. In particular, was calculated for a 5 mm layer of hydrogel spread on the inner vertical surface of a cylinder 10 × 15 cm (trap-like configuration), assuming the following: (a) hydrogel density as the same as water; (b) gel spread uniformly on the surface; (c) A_t_ is the whole hydrogel application surface. In these conditions τ = 50 Pa, then the no-flow condition is τ_0ge_ > 50 Pa. Yield stress was also evaluated in sorbitol and masonite maceration prepared samples, but no influence on yield stress behavior was found then results were not reported.

#### 2.8.6. Morphological Analysis

Scanning electronic microscope was performed through SEM EVO^®^ 40, Carl Zeiss AG on CL-HEC and CL-SA samples to evaluate the surface morphology. Energy dispersive spectroscopy (EDX, by Bruker Nano XFlash detect 5010 coupled with SEM) analyses were performed on CL-HEC hydrogels in the range 0–4 kev, creating an elemental map to evaluate the presence of possible repelling elements derived from crosslinking reaction. The analysis was performed to study the potential role of morphology and composition in repellent effect of crosslinked hydrogels.

### 2.9. Statistical Analysis

Ordinary one-way and two-way ANOVA analyses were performed using Graphpad Prism 8 software to investigate the effect of different polymer types and concentrations, salinity, pH, sorbitol concentration, and turbidity on the oviposition behavior of *Aedes Albopictus* (expressed through T/C). The analyses of variance were followed by Tukey’s or Bonferroni’s post hoc multiple comparison test. Parameters employed in ANOVA analysis were *p* < 0.05 at 95% level of confidence. All statistical analyses cited in the text will be reported in Appendix A.

## 3. Results

### 3.1. Oviposition Assays on Ades albopictus

#### 3.1.1. Oviposition Assay 1: Proof of Concept, Effects of Hydrogel Type, and Polymer Concentration

Physical hydrogels (HEC2-30 and SA2-30) and crosslinked hydrogels (CL-HEC2-30 and CL-SA2-30) were tested.

A two-way ANOVA was performed to analyze the effect of oviposition substrate type and concentration on oviposition preference (T/C) in crosslinked and physical hydrogels.

The assay proved that physical hydrogels can be employed as oviposition substrates. Polymer concentration and substrate had a statistically significant effect on T/C (respectively F (7, 32) = 7.933, *p* < 0.0001; F (7, 32) = 79.96, *p* < 0.0001; F (1, 32) = 46.12, *p* < 0.0001).

**Figure 2:**
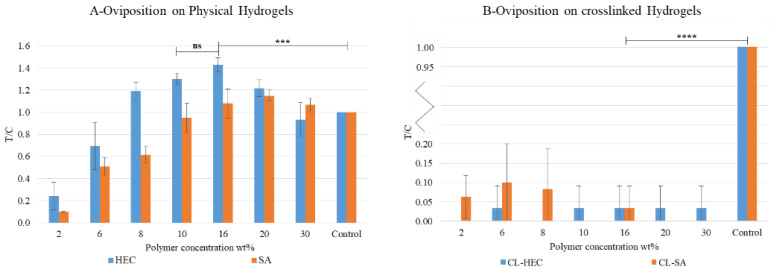
Absorbing paper was used as control. T/C is the ratio between eggs laid on the sample (T) and control (C). All the tests were carried at 25 °C and 80% relative humidity (RH) pH = 6.5; Salinity < 1%. Statistical analysis legend: *p* > 0.0123 = not significant (ns); *p* < 0.0002 = ***; *p* < 0.0001 = ****.

T/C linearly increases in the concentration range HEC2-16 and SA2-20 (evaluated using Pearson’s correlation coefficient only in the subsets HEC2-16 and SA2-20 R^2^_HEC2-16_ = 0.819 and R^2^_SA2-20_ = 0.875, Table A4). T/C > 1 for HEC8-20 and SA16-30, reaching the global maximum at HEC16 (T/C = 1.43, Figure 2A), then it decreases, becoming less attractive than control at HEC30. However, Tuckey’s multiple comparison test showed that HEC16 registered the highest statistically significant T/C value compared to the control. (Table A1 and Table A2, Figure 2A). Consequently, HEC16 was selected as the best performing oviposition substrate in oviposition assay 1.

On the contrary, the analysis revealed no statistically significant interaction or effect of the polymer type in CL-Hydrogels, and generally, a significant preference for the control over polymeric substrates emerged. The T/C in crosslinked formulations was close to 0. Then they showed a repellent effect (Table A3, Figure 2B).

#### 3.1.2. Oviposition Assay 2: Effects of Salinity, pH, Sorbitol, and Turbidity on Oviposition

One-way ANOVA analysis showed that the effect of salinity, pH, sorbitol concentration and turbidity on T/C was statistically significant (respectively F (5, 12) = 132.1, *p* < 0.0001; F (6, 14) = 66.89, *p* < 0.0001; F (6, 14) = 16.33, *p* < 0.0001; F (6, 14) = 80.01, *p* < 0.0001; Table A5, Table A6, Table A7 and Table A8).

**Figure 3:**
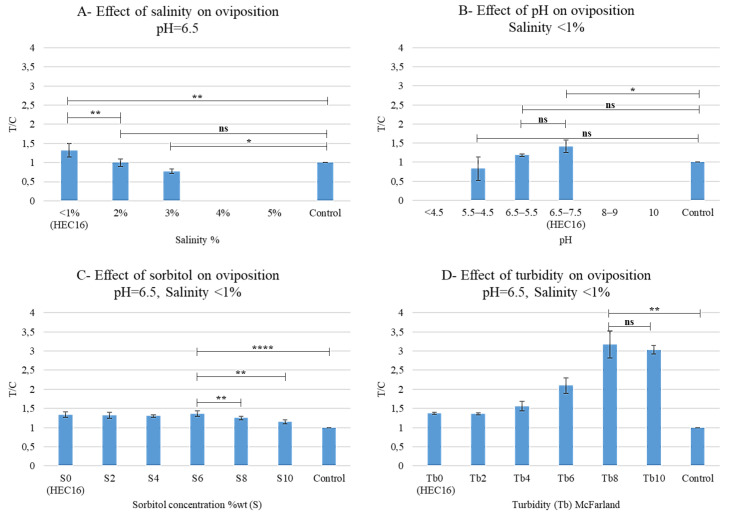
Oviposition assay 2: Effects on oviposition preferences of salinity (**A**), pH (**B**), Sorbitol concentration, S (**C**), and turbidity Tb (**D**). Paper was used as control. T/C is the ratio between eggs laid on sample (T) and control (C). All the tests were carried at 25 °C and 80% of relative humidity (RH). Statistical analysis legend: *p* > 0.0123 = not significant (ns); *p* < 0.033 = *; *p* < 0.0021 = **; *p* < 0.0001 = ****.

In particular, no oviposition was reported on the substrates having salinity > 3%, pH > 7.5, and pH < 4.5, while the Tuckey’s post hoc test confirmed that the preference for the samples having salinity < 1% and 6.5 < pH < 7.5 (they correspond to HEC16) was still statistically significant (Figure 3A,B, Table A5 and Table A6), then they were considered as the best performing substrates.

No evident preference or dislike for a specific sorbitol concentration emerged (Figure 3C). Then the highest possible sorbitol concentration could be added to the formulation as a humectant. However, S6 was considered the most suitable sorbitol concentration because it registered the highest statistically significant T/C value compared to the control (T/C = 1.37, Figure 3C and Table A7). When S8-10 concentrations were related to a statistically significant decrease in T/C (Figure 3C and Table A7), then they were not preferred as sorbitol concentrations.

Turbidity had a great impact on oviposition preference. In particular, Tb = 8 was selected as the best performing because it registered the highest statistically significant T/C value compared to the control (T/C = 3.1, Figure 3D, Table A8).

#### 3.1.3. Oviposition Assay 3: Comparison between Best Performing Formulation over Two Weeks

A two-way ANOVA (Table A9) was performed to analyze the effect of oviposition substrate and time (weeks) on oviposition preference in terms of T/C. The two-way ANOVA revealed that there was a statistically significant interaction between the effects of substrate and time (F (4, 20) = 12.68, *p* < 0.0001). Simple main effects analysis showed that substrate and time have a statistically significant effect on T/C (*p* < 0.0001 and *p* = 0.0020, Table A9).

**Figure 4:**
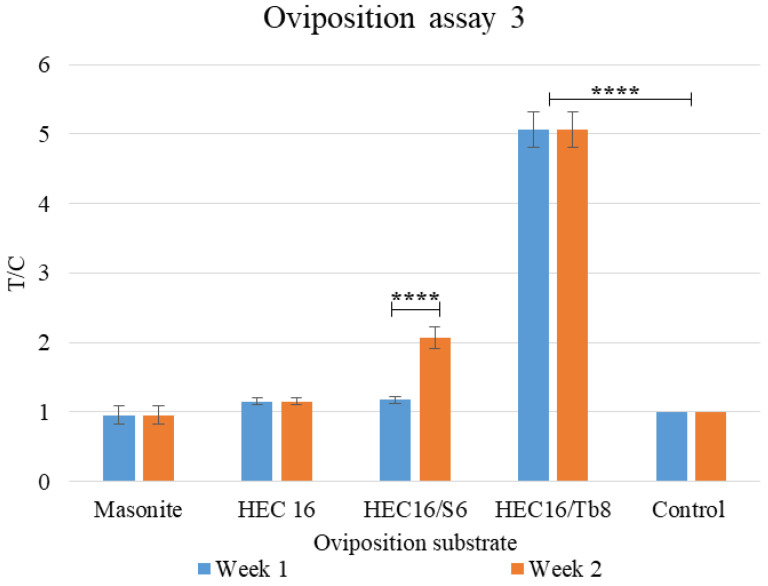
Oviposition assay 3: Substrate preference between masonite and all best performing gels in oviposition assay 2. HEC16 represents the best performing in pH and salinity assays, pH = 6.5, and salinity < 1%. Absorbing paper was used as control. T/C = eggs on sample (T)/eggs on control (C, absorbing paper). All the tests were carried out at 25 °C and 80% of relative humidity (RH). Statistical analysis legend: *p* < 0.0001 = ****.

Tuckey’s multiple comparison test (Figure 4, Table A9) showed that during the first week, HEC16/Tb8 samples registered the highest statistically significant T/C value (T/C = 5.1), confirming the positive impact of this specific turbidity value on oviposition substrate selection. By comparing weeks 1 and 2, HEC16/Tb8 remained the preferred substrate, but only HEC16/S6 registered an increase in oviposition (T/C_WEEK1_ = 1.2, T/C_WEEK1_ = 2.1, Figure 4, Table A9). The increase in HEC16/S6 can be attributed to sorbitol’s humectant action. Sorbitol improves water retention inside the substrate, making it attractive for a longer period compared to masonite, control, and the other hydrogel substrates.

Consequently, we considered the HEC16/S6/Tb8 as the best performing substrate formulation at the end of the oviposition test. It will combine the luring effect shown by the Tb8 formulation and the sorbitol’s humectant effect.

#### 3.1.4. On Field-Oviposition Assay on Best-Performing Hydrogel Formulation over 30 Days

A two-way ANOVA (Table A10) was performed to analyze the effect of oviposition substrate and time (days) on oviposition preference when used in field conditions inside cardboard-made traps (HEC16 and HEC16/S6/Tb8) and a standard masonite-based trap (as control). The two-way ANOVA analysis revealed that there was a statistically significant interaction between the effects of substrate and time (F (2, 12) = 309.2, *p* < 0.0001). Simple main effects analysis showed that substrate and time have a statistically significant effect on T/C (*p* < 0.0001; *p* < 0.0001, Table A10).

**Figure 5:**
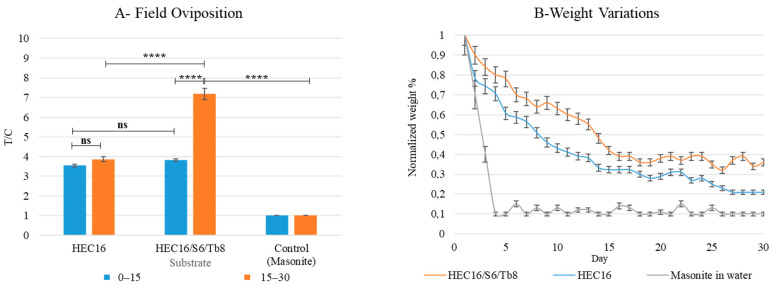
(**A**) Oviposition within 30 days using cardboard commercial trap (Easy trap by Gea srl), (**B**) Water evaporation in field conditions monitored through weight variation. Control is a standard ovitrap (masonite in distilled water in a black jar). T/C = eggs on sample (T)/eggs on control (C). Environmental conditions were 23 ± 1 °C, 75% RH (data from Fondazione Osservatorio Meterologico Milano Duomo). All the samples have pH = 6.5 and salinity < 1%. Statistical analysis legend: *p* > 0.0123 = not significant (ns); *p* < 0.0001 = ****.

Tuckey’s multiple comparison test (Figure 5A, Table A10) showed that during the period 0–15 days, the trap containing HEC16/S6/Tb8 substrate registered the highest statistically significant T/C value (T/C = 3.8). The preference for HEC16/S6/Tb8 compared to HEC16 within 0–15 days was not statistically relevant (Figure 5A, Table A10), even though they were preferred over masonite traps (*p* < 0.0001, Table A10). During the period of 0–15 days, HEC16 has an efficacy similar to HEC17/S6/Tb8. This could be attributed to a faster water release than HEC17/S6/Tb8, which could make the substrate more detectable by insects. The advantage is evidently lost in the 15–30 days interval when the water content decreases excessively (initial weights were reduced to 36% and between 21 and 10% after 30, Figure 5B). In fact, during the period of 15–30 days, there was a two-fold increase in oviposition on HEC16/S6/Tb8 (T/C = 7.18 vs. T/C = 3.8), whereas no significant T/C variations were registered in HEC16 and masonite (Figure 5A and Table A10). Weight oscillation values can be related to hydrogel and cardboard hygroscopic behavior, which can rescue water from environmental humidity (especially during rainy days).

### 3.2. Substrates Characterization

#### 3.2.1. Calorimetric Analysis of Free Water Content

**Figure 6:**
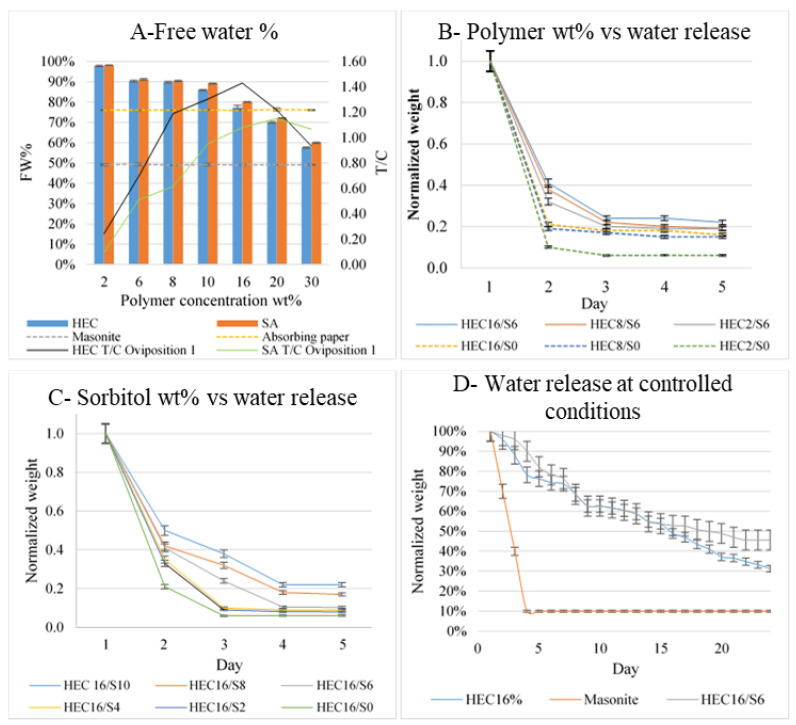
(**A**) Free water % values in hydrogel samples and controls compared to oviposition values curves. Water release measurement expressed through weight values variations with time: (**B**) Water release vs. Polymer and Sorbitol wt% and (**C**) Water release vs. Sorbitol wt%. (**D**) Water release at controlled conditions in climatic chamber 25 °C, 80% RH. All results are expressed as value ± SD.

Free water % is inversely proportional to polymer concentration (Figure 6A) regardless of the polymer. HEC and SA hydrogels have almost the same free water amount with equal polymer concentration, whereas absorbing paper and masonite contain about 80% and 50% of free water, respectively. Comparing HEC and SA oviposition values registered during oviposition assay 1 to FW%, no correlation emerged between them. For example, HEC16, SA16, and absorbing paper presented almost the same FW% but different T/C, leading to the hypothesis that substrate texture or water evaporation rate parameters are more impactful than the simple water content.

#### 3.2.2. Effect of Sorbitol and Polymer Concentration on Water Release at Controlled T and RH

Weight loss due to free water evaporation and evaporation rate at 25 °C and 80% RH (slope of the segment between days 1 and 2) was affected by the sorbitol and polymer concentration. The samples with the highest sorbitol and polymer concentrations presented the lowest evaporation rates and the highest final weight after 5 days at controlled conditions (e.g., HEC2-16 < HEC2-16/S6 and HEC16/S10 > HEC16/S2-8, Figure 6B,C). Consequently, sorbitol and polymer concentration affected water retention and release in oviposition substrates, two aspects that could have a role in the oviposition preference of tiger mosquitoes.

#### 3.2.3. Water Release in Field-like Conditions at Controlled T and RH

Weight loss and evaporation rate in trap setup (Figure 6D) followed the same trend already previously described in Section 3.2.2. Evaporation in HEC16/S6 was slower than in HEC16, and masonite was almost dry after 4 days. After 24 days in HEC16, HEC16/S6, and masonite, final residual weights were 45%, 31%, and 10% of the initial weight, and DSC free water analysis performed as described above revealed 25%, 18% and no residual of free water. Lower residual water found in field-tested samples (Figure 5B) compared to traps tested at controlled conditions can be attributed to the different geometries of traps employed. Consequently, since water presence is essential for oviposition, it is possible to conclude that hydrogels, particularly sorbitol formulations, can extend substrate activity inside a trap for at least 24 days.

#### 3.2.4. Gel Viscosity and Yield Stress Measurement

**Figure 7:**
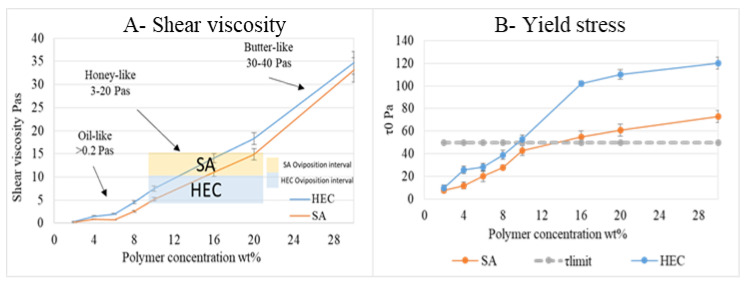
(**A**) Shear viscosity values ± SD for different HEC and SA concentrations (wt%) evaluated in a single shear rate test (50 s^−1^) at 25 °C in plate-plate configuration. (**B**) Yield stress values ± SD with polymer concentration evaluated in a shear rate range 0–100 s^−1^ at 25 °C with plate-plate configuration.

Shear viscosity is the parameter used to describe the substrate texture. Viscosity increases with polymer concentration, Figure 7A, then polymer concentration affects texture.

From the comparison between viscosity and oviposition in physical hydrogels, it emerged that oil-like compositions (e.g., shear viscosity > 0.2 Pas [66]) obtained lower T/C levels (<1) and HEC16-20 and SA16-20 concentrations (honey-like, 11–18 Pas [67]) resulted as the most attractive. A further increase in polymer concentration (butter-like, shear viscosity > 20 Pas [68]) leads to a decrease in T/C.

Yield stress values reported in Figure 7B, increase with polymer concentration. It becomes higher than the threshold value (50 Pa, previously calculated) for HEC10. Yield stress seems to not influence oviposition (T/C > 1 at HEC8 for yield stress = 39 ± 4.1 Pa), but it guarantees that physical hydrogels can be spread without flowing (as it happens in the trap setup experiment).

#### 3.2.5. Morphological Analysis

**Figure 8:**
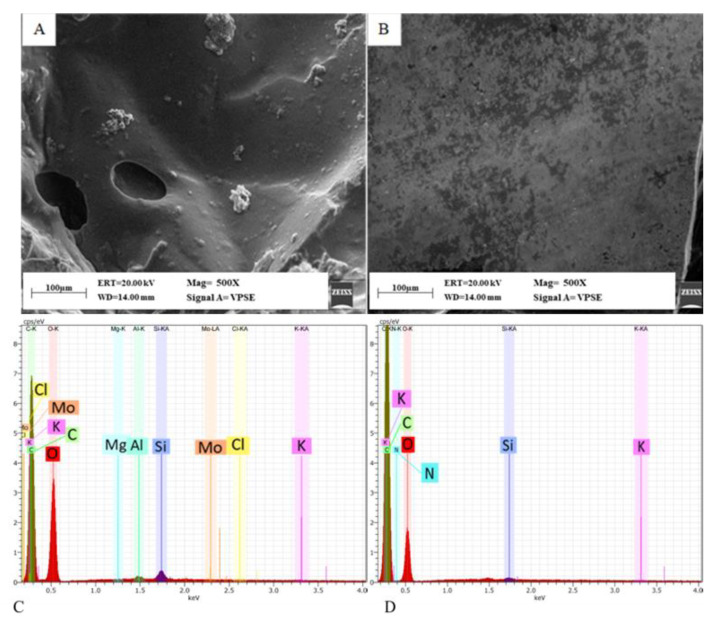
SEM micrographs (scale bar 100 µm, Mag = 500×): (**A**) CL-HEC8 and (**B**) CL-SA8 samples as examples of crosslinked hydrogel surface. EDX analysis 0–4 kev on (**C**) CL-HEC8 and (**D**) CL-SA8 samples.

SEM analysis and EDX were conducted on CL-HEC and CL-SA samples. SEM micrographs revealed the presence of smooth surfaces with micrometric pores or no pores (Figure 8A,B) and salt clusters (Figure 8A). EDX analysis showed the presence of Cl in CL-HEC samples (Figure 8C). The Cl presence can be attributed to the production process of the CL-HEC samples. In particular, Cl is a residue of the NaOH neutralization process carried out using HCl. Differently, no residual elements due to the crosslinking process were found in CL-SA samples (Figure 8D). Smooth surface samples and Cl could have been repellent factors for *Aedes albopictus* oviposition [78,79].

## 4. Discussion

The main purpose of the research was to use biopolymer-based hydrogels to produce a biomimetic substrate capable of replicating the main attractive features of an oviposition substrate for the tiger mosquito, as proposed by the proposed approach called biomimetic lure and kill. This approach hypothesized that such a material could be more attractive than standard oviposition substrates (e.g., absorbing paper and masonite). It also could have an application advantage as it is able to make the ovitraps (in which it is intended to be used) more attractive and for a longer period as it is able to dehydrate more slowly than common deposition substrates. For this purpose, a wide panel of physical and crosslinked hydrogel concentrations were tested due to the extreme variety of substrates used by the tiger mosquito to lay their eggs. Soft (e.g., mud-like) to very rigid and rough (e.g., wooden-like) oviposition substrates were tested and the effects on oviposition preferences of pH, salinity, water release (through different sorbitol concentrations), and turbidity were evaluated [58,66].

It was observed that the ovipositing females preferred physical hydrogels to control, whereas the crosslinked hydrogels showed a repellent effect (almost no oviposition), probably due to smooth surfaces and Cl residuals from the crosslinking process, which are already known as deterrents for *Aedes albopictus* oviposition [60,78,79,80]. In particular, it was found that a 2-Hydroxyethylcellulose-based hydrogel formulation (HEC16) containing 16 wt% of polymer can attract ovipositing tiger mosquitoes more than control. In particular, the hydrogel substrate HEC16 has pH = 6.5–7.5, salinity < 1%, 6 wt% of sorbitol concentration, and turbidity of 8 McF (HEC16/S6/Tb8) collected 5 times more eggs than masonite in a lab oviposition assay. When HEC16/S6/Tb8 was employed in a low-cost cardboard trap in field conditions, the device resulted in seven times more capturing than a standard masonite-based ovitrap. Furthermore, the formulation HEC16/S6/Tb8 was able to extend up to four weeks rather than one week the active period of the traps, confirming what was claimed in the BL&K-TM approach.

Downstream of oviposition assays, it was possible to identify an attractive range for all the parameters tested and a specific value to define a preferred formulation, as reported in Table 2.

Some oviposition drivers have a direct influence on increasing the number of eggs laid. Among them, turbidity showed the greatest impact on oviposition preference. Oviposition dependence from turbidity could be related both to a higher organic presence in water used to produce hydrogels (as already reported for water in breeding sites [81]) or to a wood-like color better simulating a natural oviposition surface (e.g., tree holes, tree bark, etc.). Masonite’s role as an olfactory stimulant might even be supposed in oviposition assay 2, but the higher oviposition in HEC16, HEC16/S6 (hydrogels without masonite maceration) over masonite stick registered in oviposition assay 3 allows excluding it.

Other parameters, such as salinity% or pH, do not show a deposition-promoting effect, but within some ranges, have a repellent effect. Salinity ≥ 2% and 4.5 ≤ pH ≤ 5.5 caused a mild decrease in the HEC16 luring effect (T/C ≈ 1), whereas there was a clear and statistically significant absence of eggs for salinity > 3%, pH > 7.5, and pH < 4.5. As reported in the literature, a possible explanation is that extreme pH and salinity harm *Aedes albopictus* tactile and olfactory senses [82], leading to a repellent effect.

Numerous properties that can impact oviposition preferences, such as texture, free water amount, and water release, are polymer concentration-dependent [83]. Consequently, they can be easily adjusted by changing the polymer type or concentration. For example, substrate texture seems to have a greater effect on the choice of the substrate than the quantity of free water (FW%) and the evaporation rate. This is demonstrated by the preference for honey-like over oil-like textures, although the latter are less viscous, and they should be more easily identifiable as they evaporate faster (water vapor is commonly a powerful signal for tiger mosquitoes to identify a potential oviposition site [68]). However, an excessive reduction in evaporation rate can reduce oviposition. That is what happens for HEC20-30 and HEC16/S8-10, where T/C reduction could be related to an excessive reduction in evaporation rate due to polymer or sorbitol concentration [67,73,84]. At the same time, the presence of sorbitol was essential to extending the active period of the trap both in the laboratory and in the field, so it is useful within the formulation.

It emerges that oviposition is a multifactorial phenomenon and numerous aspects must be balanced to obtain at the same time a biomimetic luring substrate and a technically advantageous product. It follows that an insect-custom-made product, designed and optimized to match the features of a particular insect, can be more attractive and cost-effective than a generic one. Therefore, the biomimetic approach could be useful for implementing lure and kill strategies not only for tiger mosquitoes but for a larger panel of pests.

In that specific case of the deposition of the tiger mosquito, HEC16/S6/Tb8 probably was more attractive than the standard substrates because it is able to get closer to the ideal deposition conditions reported in the literature, and also because it does not present repellent conditions, differently from CL-hydrogels.

In conclusion, in addition to the lure advantages due to the biomimetic design of the substrate, the research gave an initial proof that hydrogels could guarantee a technical solution to the problems of duration, maintenance, and disposal of the traps and associated costs. In particular, when applied to biodegradable and low-cost traps (e.g., made of PLA) or cardboard-made supports (similar to glue-based sticky traps [51,53,85,86]), due to the lack of liquid water in the trap, hydrogel oviposition substrates can be a valuable commercial solution. Furthermore, they can be stored after lyophilization (e.g., through freeze-drying) and subsequently rehydrated (without loss of properties), ensuring ease of use, storage, and transport [87]. This use could ensure a possible industrial scale-up and a low-cost and easily storable final product.

At the same time, these natural hydrogels can be exploited to support the survival and growth of biopesticides [28]. The only constraint is an overlapping between the proliferation condition of the biocide needed and the attractiveness of the substrate for the mosquito. Verifying the possibility of hosting the living bioinsecticide represents the next research step in BL&K validation.

Another possible direction to explore for future studies is the possibility to obtain a mechanical insecticide action (i.e., due only to the material action, e.g., by varying its viscosity) to eliminate the need for the biocide, then increase the safety of the devices and potentially remove the costs for biocide registration [88]. More targeted studies and experimental setups must be carried out to identify other oviposition driving parameters to be replicated through a specific hydrogel design. Furthermore, it is necessary to progress in the knowledge of breeding sites, possibly through the use of sensors for live monitoring of the oviposition behavior in different conditions, in order to refine the design of the substrate, making it more and more able to reproduce the ideal oviposition conditions.

## 5. Conclusions

The work proved that hydrogels could attract tiger ovipositing mosquitoes more than a simple standard substrate, i.e., confirmed the first hypothesis of the BL&K-TM approach. It was identified as a 2-Hydroxyethylcellulose-based hydrogel formulation (HEC16) containing 16 wt% of polymer that collected more eggs than the control.

By evaluating the effects on oviposition of pH, salinity, sorbitol concentration, and turbidity, it was found another formulation (HEC16/S6/Tb8) having pH = 6.5–7.5, salinity < 1%, 6% of sorbitol by weight, and turbidity (Tb)=8 McF. When HEC16/S6/Tb8 was applied to a low-cost cardboard trap in field conditions, the device resulted in seven times more capturing than a standard masonite-based ovitrap. Furthermore, the formulation HEC16/S6/Tb8 was able to extend up to four weeks rather than one week the active period of the traps, confirming what was claimed in the BL&K-TM approach.

## 6. Patents

The product has been patented. Italian patent N. 102019000018065.

## Figures and Tables

**Table 1:** A panel of possible oviposition influencing parameters and possible suitable conditions reported in literature.

Oviposition Parameters							
Parameters	pH	Salinity [%]	Substrate Composition	Water Content [wt%]	Substrate Texture/Orientation	Morphology	Turbidity
**Suitable conditions**	From mild acidic to basic	Mild	Organic	>0%	From mud to wood-like/Sloped	Rough	Cloudy water

**Table 2:** A panel of range for oviposition parameters and substrate composition identified through tests.

Oviposition Parameters								
pH	Salinity [%]	Turbidity [McF]	Sorbitol [wt%]	Composition [wt%]	Water Content [wt%]	Viscosity [Pas]	Yield Stress [Pa]	Morphology
**Working range**								
4.5–7.5	<3%	0–10	0–10	HEC8-20/SA16-30	>0%	4.5–18.3/11–33.1	>50	Rough
**Best preparation**								
6.5–7.5	<1%	8	6	HEC16/S6/Tb8	80%	14.1	>50	Rough

## Data Availability

The data that support the findings of this study are available from the corresponding author upon request.

## Appendix A

**Table A1:** Ordinary two-way oviposition ANOVA analysis of HEC2-30 and SA2-30 concentrations and Tukey’s multiple comparisons test to evaluate the effect of. *p* > 0.0123 = not significant (ns); *p* < 0.033 = *; *p* < 0.0021 **; *p* < 0.0002 ***; *p* < 0.0001 = ****.

Ordinary Two-Way ANOVA						
**Alpha**	0.05					
**Source of Variation**	% of total variation	*p* value	*p* value summary			
**Interaction**	8.009	<0.0001	****			
**Polymer concentration**	80.72	<0.0001	****			
**Substrate**	6.652	<0.0001	****			
**ANOVA table**						
	SS	DF	MS	F (DFn. DFd)	*p* value	
**Interaction**	0.5354	7	0.07649	F (7, 32) = 7.933	*p* < 0.0001	
**Polymer concentration**	5.396	7	0.7709	F (7, 32) = 79.96	*p* < 0.0001	
**Substrate**	0.4447	1	0.4447	F (1, 32) = 46.12	*p* < 0.0001	
**Residual**	0.3085	32	0.009642			
**Tukey’s Multiple Comparisons Test**						
**Number of families**		2				
**Number of comparisons per family**		28				
**Alpha**		0.05				
		Mean Diff.	95% CI of diff.	Summary	Adjusted *p* Value	
**HEC**						
**2% vs. 6%**		**−0.45**	**−0.7097 to −0.1903**	********	**<0.0001**	
**2% vs. 8%**		**−0.9467**	**−1.206 to −0.6870**	********	**<0.0001**	
**2% vs. 10%**		**−1.06**	**−1.320 to −0.8003**	********	**<0.0001**	
**2% vs. 16%**		**−1.187**	**−1.446 to −0.9270**	********	**<0.0001**	
**2% vs. 20%**		**−0.9733**	**−1.233 to −0.7136**	********	**<0.0001**	
**2% vs. 30%**		**−0.69**	**−0.9497 to −0.4303**	********	**<0.0001**	
**2% vs. control**		**−0.7567**	**−1.016 to −0.4970**	********	**<0.0001**	
**6% vs. 8%**		**−0.4967**	**−0.7564 to −0.2370**	********	**<0.0001**	
**6% vs. 10%**		**−0.61**	**−0.8697 to −0.3503**	********	**<0.0001**	
**6% vs. 16%**		**−0.7367**	**−0.9964 to −0.4770**	********	**<0.0001**	
**6% vs. 20%**		**−0.5233**	**−0.7830 to −0.2636**	********	**<0.0001**	
**6% vs. 30%**		**−0.24**	**−0.4997 to 0.01971**	**ns**	**0.0869**	
**6% vs. control**		**−0.3067**	**−0.5664 to −0.04696**	*****	**0.0118**	
**8% vs. 10%**		**−0.1133**	**−0.3730 to 0.1464**	**ns**	**0.8443**	
**8% vs. 16%**		**−0.24**	**−0.4997 to 0.01971**	**ns**	**0.0869**	
**8% vs. 20%**		**−0.02667**	**−0.2864 to 0.2330**	**ns**	**>0.9999**	
**8% vs. 30%**		**0.2567**	**−0.003039 to 0.5164**	**ns**	**0.0546**	
**8% vs. control**		**0.19**	**−0.06971 to 0.4497**	**ns**	**0.2897**	
**10% vs. 16%**		**−0.1267**	**−0.3864 to 0.1330**	**ns**	**0.7583**	
**10% vs. 20%**		**0.08667**	**−0.1730 to 0.3464**	**ns**	**0.9563**	
**10% vs. 30%**		**0.37**	**0.1103 to 0.6297**	******	**0.0014**	
**10% vs. control**		**0.3033**	**0.04363 to 0.5630**	*****	**0.0131**	
**16% vs. 20%**		**0.2133**	**−0.04637 to 0.4730**	**ns**	**0.1719**	
**16% vs. 30%**		**0.4967**	**0.2370 to 0.7564**	********	**<0.0001**	
**16% vs. control**		**0.43**	**0.1703 to 0.6897**	*******	**0.0002**	
**20% vs. 30%**		**0.2833**	**0.02363 to 0.5430**	*****	**0.0247**	
**20% vs. control**		**0.2167**	**−0.04304 to 0.4764**	**ns**	**0.1586**	
**30% vs. control**		**−0.06667**	**−0.3264 to 0.1930**	**ns**	**0.9898**	
**SA**						
**2% vs. 6%**		**−0.4133**	**−0.6730 to −0.1536**	*******	**0.0003**	
**2% vs. 8%**		**−0.52**	**−0.7797 to −0.2603**	********	**<0.0001**	
**2% vs. 10%**		**−0.8533**	**−1.113 to −0.5936**	********	**<0.0001**	
**2% vs. 16%**		**−0.9833**	**−1.243 to −0.7236**	********	**<0.0001**	
**2% vs. 20%**		**−1.053**	**−1.313 to −0.7936**	********	**<0.0001**	
**2% vs. 30%**		**−0.97**	**−1.230 to −0.7103**	********	**<0.0001**	
**2% vs. control**		**−0.9033**	**−1.163 to −0.6436**	********	**<0.0001**	
**6% vs. 8%**		**−0.1067**	**−0.3664 to 0.1530**	**ns**	**0.8803**	
**6% vs. 10%**		**−0.44**	**−0.6997 to −0.1803**	*******	**0.0001**	
**6% vs. 16%**		**−0.57**	**−0.8297 to −0.3103**	********	**<0.0001**	
**6% vs. 20%**		**−0.64**	**−0.8997 to −0.3803**	********	**<0.0001**	
**6% vs. 30%**		**−0.5567**	**−0.8164 to −0.2970**	********	**<0.0001**	
**6% vs. control**		**−0.49**	**−0.7497 to −0.2303**	********	**<0.0001**	
**8% vs. 10%**		**−0.3333**	**−0.5930 to −0.07363**	******	**0.0049**	
**8% vs. 16%**		**−0.4633**	**−0.7230 to −0.2036**	********	**<0.0001**	
**8% vs. 20%**		**−0.5333**	**−0.7930 to −0.2736**	********	**<0.0001**	
**8% vs. 30%**		**−0.45**	**−0.7097 to −0.1903**	********	**<0.0001**	
**8% vs. control**		**−0.3833**	**−0.6430 to −0.1236**	*******	**0.0009**	
**10% vs. 16%**		**−0.13**	**−0.3897 to 0.1297**	**ns**	**0.7343**	
**10% vs. 20%**		**−0.2**	**−0.4597 to 0.05971**	**ns**	**0.2339**	
**10% vs. 30%**		**−0.1167**	**−0.3764 to 0.1430**	**ns**	**0.8245**	
**10% vs. control**		**−0.05**	**−0.3097 to 0.2097**	**ns**	**0.9983**	
**16% vs. 20%**		**−0.07**	**−0.3297 to 0.1897**	**ns**	**0.9865**	
**16% vs. 30%**		**0.01333**	**−0.2464 to 0.2730**	**ns**	**>0.9999**	
**16% vs. control**		**0.08**	**−0.1797 to 0.3397**	**ns**	**0.9714**	
**20% vs. 30%**		**0.08333**	**−0.1764 to 0.3430**	**ns**	**0.9644**	
**20% vs. control**		**0.15**	**−0.1097 to 0.4097**	**ns**	**0.5793**	
**30% vs. control**		**0.06667**	**−0.1930 to 0.3264**	**ns**	**0.9898**	

**Table A2:** Ordinary two-way oviposition ANOVA analysis of HEC2-30 and SA2-30 concentrations and Bonferroni’s multiple comparisons test. *p* > 0.0123 = not significant (ns); *p* < 0.0002 ***; *p* < 0.0001 = ****.

Ordinary Two-Way ANOVA					
**Alpha**	0.05				
**Source of Variation**	% of total variation	*p* value	*p* value summary		
**Interaction**	8.009	<0.0001	****		
**Row Factor**	80.72	<0.0001	****		
**Column Factor**	6.652	<0.0001	****		
**ANOVA table**					
	SS	DF	MS	F (DFn. DFd)	*p* value
**Interaction**	0.5354	7	0.07649	F (7, 32) = 7.933	*p* < 0.0001
**Row Factor**	5.396	7	0.7709	F (7, 32) = 79.96	*p* < 0.0001
**Column Factor**	0.4447	1	0.4447	F (1, 32) = 46.12	*p* < 0.0001
**Residual**	0.3085	32	0.009642		
**Bonferroni’s multiple comparisons test**					
**Number of families**	1				
**Number of comparisons per family**	8				
**Alpha**	0.05				
	Mean Diff.	95% CI of diff.	Significant?	Summary	Adjusted *p* Value
**HEC-SA**					
**2%**	0.1467	−0.08802 to 0.3813	No	ns	0.6134
**6%**	0.1833	−0.05135 to 0.4180	No	ns	0.2318
**8%**	0.5733	0.3387 to 0.8080	Yes	****	<0.0001
**10%**	0.3533	0.1187 to 0.5880	Yes	***	0.0009
**16%**	0.3500	0.1153 to 0.5847	Yes	***	0.0010
**20%**	0.06667	−0.1680 to 0.3013	No	ns	>0.9999
**30%**	−0.1333	−0.3680 to 0.1013	No	ns	0.8485
**Control**	0.000	−0.2347 to 0.2347	No	ns	>0.9999

**Table A3:** Ordinary two-way oviposition ANOVA analysis and Tukey’s multiple comparisons test to evaluate the effect of polymer type and concentration on T/C in the groups CL-HEC2-30 and CL-SA2-30, *p* > 0.0123 = not significant (ns); *p* < 0.0001 = ****.

Ordinary Two-Way ANOVA					
**Alpha**	0.05				
**Source of Variation**	% of total variation	*p* value	*p* value summary		
**Interaction**	0.5089	0.2648	ns		
**Concentration**	97.71	<0.0001	****		
**Substrate**	0.04771	0.3557	ns		
**ANOVA table**					
	SS	DF	MS	F (DFn. DFd)	*p* value
**Interaction**	0.02569	7	0.00367	F (7, 32) = 1.339	*p* = 0.2648
**Polymer Concentration**	4.932	7	0.7046	F (7, 32) = 257.0	*p* < 0.0001
**Substrate**	0.002408	1	0.002408	F (1, 32) = 0.8784	*p* = 0.3557
**Residual**	0.08773	32	0.002742		
**Tukey’s multiple comparisons test**					
**Number of families**	2				
**Number of comparisons per family**	28				
**Alpha**	0.05				
	**Mean Diff.**	**95% CI of diff.**	**Summary**	**Adjusted *p* Value**	
**CL-HEC**					
**2% vs. 6%**	−0.03333	−0.1718 to 0.1052	ns	0.9931	
**2% vs. 8%**	0	−0.1385 to 0.1385	ns	>0.9999	
**2% vs. 10%**	−0.03333	−0.1718 to 0.1052	ns	0.9931	
**2% vs. 16%**	−0.03333	−0.1718 to 0.1052	ns	0.9931	
**2% vs. 20%**	−0.03333	−0.1718 to 0.1052	ns	0.9931	
**2% vs. 30%**	−0.03333	−0.1718 to 0.1052	ns	0.9931	
**2% vs. control**	−1	−1.138 to −0.8615	****	<0.0001	
**6% vs. 8%**	0.03333	−0.1052 to 0.1718	ns	0.9931	
**6% vs. 10%**	−2.8 × 10^−17^	−0.1385 to 0.1385	ns	>0.9999	
**6% vs. 16%**	−5.6 × 10^−17^	−0.1385 to 0.1385	ns	>0.9999	
**6% vs. 20%**	0	−0.1385 to 0.1385	ns	>0.9999	
**6% vs. 30%**	8.33 × 10^−17^	−0.1385 to 0.1385	ns	>0.9999	
**6% vs. control**	−0.9667	−1.105 to −0.8282	****	<0.0001	
**8% vs. 10%**	−0.03333	−0.1718 to 0.1052	ns	0.9931	
**8% vs. 16%**	−0.03333	−0.1718 to 0.1052	ns	0.9931	
**8% vs. 20%**	−0.03333	−0.1718 to 0.1052	ns	0.9931	
**8% vs. 30%**	−0.03333	−0.1718 to 0.1052	ns	0.9931	
**8% vs. control**	−1	−1.138 to −0.8615	****	<0.0001	
**10% vs. 16%**	−2.8 × 10^−17^	−0.1385 to 0.1385	ns	>0.9999	
**10% vs. 20%**	2.78 × 10^−17^	−0.1385 to 0.1385	ns	>0.9999	
**10% vs. 30%**	1.11 × 10^−16^	−0.1385 to 0.1385	ns	>0.9999	
**10% vs. control**	−0.9667	−1.105 to −0.8282	****	<0.0001	
**16% vs. 20%**	5.55 × 10^−17^	−0.1385 to 0.1385	ns	>0.9999	
**16% vs. 30%**	1.39 × 10^−16^	−0.1385 to 0.1385	ns	>0.9999	
**16% vs. control**	−0.9667	−1.105 to −0.8282	****	<0.0001	
**20% vs. 30%**	8.33 × 10^−17^	−0.1385 to 0.1385	ns	>0.9999	
**20% vs. control**	−0.9667	−1.105 to −0.8282	****	<0.0001	
**30% vs. control**	−0.9667	−1.105 to −0.8282	****	<0.0001	
**CL-SA**					
**2% vs. 6%**	−0.03667	−0.1752 to 0.1018	ns	0.9878	
**2% vs. 8%**	−0.02	−0.1585 to 0.1185	ns	0.9997	
**2% vs. 10%**	0.06333	−0.07515 to 0.2018	ns	0.8113	
**2% vs. 16%**	0.03	−0.1085 to 0.1685	ns	0.9963	
**2% vs. 20%**	0.06333	−0.07515 to 0.2018	ns	0.8113	
**2% vs. 30%**	0.06333	−0.07515 to 0.2018	ns	0.8113	
**2% vs. control**	−0.9367	−1.075 to −0.7982	****	<0.0001	
**6% vs. 8%**	0.01667	−0.1218 to 0.1552	ns	>0.9999	
**6% vs. 10%**	0.1	−0.03849 to 0.2385	ns	0.3047	
**6% vs. 16%**	0.06667	−0.07182 to 0.2052	ns	0.7698	
**6% vs. 20%**	0.1	−0.03849 to 0.2385	ns	0.3047	
**6% vs. 30%**	0.1	−0.03849 to 0.2385	ns	0.3047	
**6% vs. control**	−0.9	−1.038 to −0.7615	****	<0.0001	
**8% vs. 10%**	0.08333	−0.05515 to 0.2218	ns	0.5295	
**8% vs. 16%**	0.05	−0.08849 to 0.1885	ns	0.9348	
**8% vs. 20%**	0.08333	−0.05515 to 0.2218	ns	0.5295	
**8% vs. 30%**	0.08333	−0.05515 to 0.2218	ns	0.5295	
**8% vs. control**	−0.9167	−1.055 to −0.7782	****	<0.0001	
**10% vs. 16%**	−0.03333	−0.1718 to 0.1052	ns	0.9931	
**10% vs. 20%**	0	−0.1385 to 0.1385	ns	>0.9999	
**10% vs. 30%**	1.39 × 10^−16^	−0.1385 to 0.1385	ns	>0.9999	
**10% vs. control**	−1	−1.138 to −0.8615	****	<0.0001	
**16% vs. 20%**	0.03333	−0.1052 to 0.1718	ns	0.9931	
**16% vs. 30%**	0.03333	−0.1052 to 0.1718	ns	0.9931	
**16% vs. control**	−0.9667	−1.105 to −0.8282	****	<0.0001	
**20% vs. 30%**	1.39 × 10^−16^	−0.1385 to 0.1385	ns	>0.9999	
**20% vs. control**	−1	−1.138 to −0.8615	****	<0.0001	
**30% vs. control**	−1	−1.138 to −0.8615	****	<0.0001	

**Table A4:** Correlation using Pearson’s correlation coefficient analysis between polymer concentration and T/C for HEC2-16 and SA2-20. *p* < 0.033 = *; *p* < 0.0021 = **.

Pearson r		
**r**	0.9053	0.9357
**95% confidence interval**	0.1144 to 0.9938	0.5160 to 0.9931
**R squared**	0.8196	0.8756
***p*** **value**		
***p*** **(two-tailed)**	0.0345	0.0061
***p*** **value summary**	*	**
**Significant? (alpha = 0.05)**	Yes	Yes
**Number of XY Pairs**	5	6

**Table A5:** One-way ANOVA analysis and Tukey’s multiple comparisons test to evaluate the effect of different salinity values on T/C. *p* > 0.0123 = not significant (ns); *p* < 0.033 = *; *p* < 0.0021 **; *p* < 0.0001 = ****.

ANOVA Summary					
**F**	132.1				
***p* value**	<0.0001				
***p* value summary**	****				
**Significant diff. among means (*p* < 0.05)?**	Yes				
**R square**	0.9822				
**ANOVA table**					
	SS	DF	MS	F (DFn. DFd)	*p* value
**Treatment (between columns)**	4.631	5	0.9262	F (5, 12) = 132.1	*p* < 0.0001
**Residual (within columns)**	0.08413	12	0.007011		
**Total**	4.715	17			
**Number of families**	1				
**Number of comparisons per family**	15				
**Alpha**	0.05				
**Tukey’s multiple comparisons test**					
	Mean Diff.	95% CI of diff.		Summary	Adjusted *p* Value
**<1% (HEC16) vs. 2%**	0.3228	0.09314 to 0.5524		**	0.0051
**<1% (HEC16) vs. 3%**	0.5509	0.3212 to 0.7805		****	<0.0001
**<1% (HEC16) vs. 4%**	1.319	1.090 to 1.549		****	<0.0001
**<1% (HEC16) vs. 5%**	1.319	1.090 to 1.549		****	<0.0001
**<1% (HEC16) vs. Control**	0.3191	0.08951 to 0.5488		**	0.0056
**2% vs. 3%**	0.2281	−0.001558 to 0.4577		ns	0.0519
**2% vs. 4%**	0.9964	0.7667 to 1.226		****	<0.0001
**2% vs. 5%**	0.9964	0.7667 to 1.226		****	<0.0001
**2% vs. Control**	−0.003630	−0.2333 to 0.2260		ns	>0.9999
**3% vs. 4%**	0.7683	0.5387 to 0.9979		****	<0.0001
**3% vs. 5%**	0.7683	0.5387 to 0.9979		****	<0.0001
**3% vs. Control**	−0.2317	−0.4613 to −0.002072		*	0.0475
**4% vs. 5%**	0.000	−0.2296 to 0.2296		ns	>0.9999
**4% vs. Control**	−1.000	−1.230 to −0.7704		****	<0.0001
**5% vs. Control**	−1.000	−1.230 to −0.7704		****	<0.0001

**Table A6:** One-way ANOVA and post-hoc Tuckey’s multiple comparison to evaluate the effect of different pH values on T/C. *p* > 0.0123 = not significant (ns); *p* < 0.033 = *; *p* < 0.0021 **; *p* < 0.0001 = ****.

ANOVA Summary					
**F**	66.89				
***p* value**	<0.0001				
***p* value summary**	****				
**Significant diff. among means (*p* < 0.05)?**	Yes				
**R square**	0.9663				
**ANOVA table**					
	SS	DF	MS	F (DFn. DFd)	*p* value
**Treatment (between columns)**	6.890	6	1.148	F (6, 14) = 66.89	*p* < 0.0001
**Residual (within columns)**	0.2403	14	0.01717		
**Total**	7.130	20			
**Tukey’s multiple comparisons test**					
**Number of families**	1				
**Number of comparisons per family**	21				
**Alpha**	0.05				
	Mean Diff.	95% CI of diff.	Summary	Adjusted *p* Value	
**<4.5 vs. 5.5–4.5**	−0.8333	−1.199 to −0.4680	****	<0.0001	
**<4.5 vs. 6.5–5.5**	−1.186	−1.551 to −0.8205	****	<0.0001	
**<4.5 vs. 6.5–7.5 (HEC16)**	−1.417	−1.782 to −1.051	****	<0.0001	
**<4.5 vs. 8–9**	0.000	−0.3653 to 0.3653	ns	>0.9999	
**<4.5 vs. 10**	0.000	−0.3653 to 0.3653	ns	>0.9999	
**<4.5 vs. Control**	−1.000	−1.365 to −0.6347	****	<0.0001	
**5.5–4.5 vs. 6.5–5.5**	−0.3525	−0.7178 to 0.01281	ns	0.0618	
**5.5–4.5 vs. 6.5–7.5 (HEC16)**	−0.5833	−0.9486 to −0.2180	**	0.0013	
**5.5–4.5 vs. 8–9**	0.8333	0.4680 to 1.199	****	<0.0001	
**5.5–4.5 vs. 10**	0.8333	0.4680 to 1.199	****	<0.0001	
**5.5–4.5 vs. Control**	−0.1667	−0.5320 to 0.1986	ns	0.7086	
**6.5–5.5 vs. 6.5–7.5 (HEC16)**	−0.2309	−0.5961 to 0.1344	ns	0.3737	
**6.5–5.5 vs. 8–9**	1.186	0.8205 to 1.551	****	<0.0001	
**6.5–5.5 vs. 10**	1.186	0.8205 to 1.551	****	<0.0001	
**6.5–5.5 vs. Control**	0.1858	−0.1795 to 0.5511	ns	0.6052	
**6.5–7.5 (HEC16) vs. 8–9**	1.417	1.051 to 1.782	****	<0.0001	
**6.5–7.5 (HEC16) vs. 10**	1.417	1.051 to 1.782	****	<0.0001	
**6.5–7.5 (HEC16) vs. Control**	0.4167	0.05137 to 0.7820	*	0.0210	
**8–9 vs. 10**	0.000	−0.3653 to 0.3653	ns	>0.9999	
**8–9 vs. Control**	−1.000	−1.365 to −0.6347	****	<0.0001	
**10 vs. Control**	−1.000	−1.365 to −0.6347	****	<0.0001	

**Table A7:** One-way ANOVA analysis between different sorbitol concentrations and Tukey’s multiple comparisons test. *p* > 0.0123 = not significant (ns); *p* < 0.033 = *; *p* < 0.0021 **; *p* < 0.0002 ***; *p* < 0.0001 = ****.

ANOVA Summary					
**F**	16.33				
***p* value**	<0.0001				
***p* value summary**	****				
**Significant diff. among means (*p* < 0.05)?**	Yes				
**R square**	0.8750				
**ANOVA table**					
	SS	DF	MS	F (DFn. DFd)	*p* value
**Treatment (between columns)**	0.3405	6	0.05675	F (6, 14) = 16.33	*p* < 0.0001
**Residual (within columns)**	0.04867	14	0.003476		
**Total**	0.3892	20			
**Tukey’s multiple comparisons test**					
**Number of families**	1				
**Number of comparisons per family**	21				
**Alpha**	0.05				
	Mean Diff.	95% CI of diff.		Summary	Adjusted *p* Value
**S10 vs. S8**	0.01667	−0.1477 to 0.1810		ns	0.9998
**S10 vs. S6**	−0.2167	−0.3810 to −0.05229		**	0.0070
**S10 vs. S4**	−0.1533	−0.3177 to 0.01104		ns	0.0749
**S10 vs. S2**	−0.1733	−0.3377 to −0.008955		*	0.0358
**S10 vs. S0 (HEC16)**	−0.1900	−0.3544 to −0.02562		*	0.0191
**S10 vs. Control**	0.1500	−0.01438 to 0.3144		ns	0.0845
**S8 vs. S6**	−0.2333	−0.3977 to −0.06896		**	0.0037
**S8 vs. S4**	−0.1700	−0.3344 to −0.005622		*	0.0406
**S8 vs. S2**	−0.1900	−0.3544 to −0.02562		*	0.0191
**S8 vs. S0 (HEC16)**	−0.2067	−0.3710 to −0.04229		*	0.0102
**S8 vs. Control**	0.1333	−0.03104 to 0.2977		ns	0.1510
**S6 vs. S4**	0.06333	−0.1010 to 0.2277		ns	0.8341
**S6 vs. S2**	0.04333	−0.1210 to 0.2077		ns	0.9665
**S6 vs. S0 (HEC16)**	0.02667	−0.1377 to 0.1910		ns	0.9972
**S6 vs. Control**	0.3667	0.2023 to 0.5310		****	<0.0001
**S4 vs. S2**	−0.02000	−0.1844 to 0.1444		ns	0.9994
**S4 vs. S0 (HEC16)**	−0.03667	−0.2010 to 0.1277		ns	0.9852
**S4 vs. Control**	0.3033	0.1390 to 0.4677		***	0.0003
**S2 vs. S0 (HEC16)**	−0.01667	−0.1810 to 0.1477		ns	0.9998
**S2 vs. Control**	0.3233	0.1590 to 0.4877		***	0.0002
**S0 (HEC16) vs. Control**	0.3400	0.1756 to 0.5044		****	<0.0001

**Table A8:** One-way ANOVA analysis and Tukey’s multiple comparisons test to evaluate the effect of Turbidity on T/C. *p* > 0.0123 = not significant (ns); *p* < 0.033 = *; *p* < 0.0021 **; *p* < 0.0002 ***; *p* < 0.0001 = ****.

ANOVA Summary					
**F**	80.01				
***p* value**	<0.0001				
***p* value summary**	****				
**Significant diff. among means (*p* < 0.05)?**	Yes				
**R square**	0.9717				
**ANOVA table**					
	SS	DF	MS	F (DFn. DFd)	*p* value
**Treatment (between columns)**	13.22	6	2.204	F (6, 14) = 80.01	*p* < 0.0001
**Residual (within columns)**	0.3856	14	0.02754		
**Total**	13.61	20			
**Tukey’s multiple comparisons test**					
**Number of families**	1				
**Number of comparisons per family**	21				
**Alpha**	0.05				
	Mean Diff.	95% CI of diff.	Summary	Adjusted *p* Value	
**Tb10 vs. Tb8**	−0.1333	−0.5960 to 0.3294	ns	0.9496	
**Tb10 vs. Tb6**	0.9333	0.4706 to 1.396	***	0.0001	
**Tb10 vs. Tb4**	1.473	1.011 to 1.936	****	<0.0001	
**Tb10 vs. Tb2**	1.673	1.211 to 2.136	****	<0.0001	
**Tb10 vs. Tb0 (HEC16)**	1.657	1.194 to 2.119	****	<0.0001	
**Tb10 vs. Control**	2.033	1.571 to 2.496	****	<0.0001	
**Tb8 vs. Tb6**	1.067	0.6040 to 1.529	****	<0.0001	
**Tb8 vs. Tb4**	1.607	1.144 to 2.069	****	<0.0001	
**Tb8 vs. Tb2**	1.807	1.344 to 2.269	****	<0.0001	
**Tb8 vs. Tb0 (HEC16)**	1.790	1.327 to 2.253	****	<0.0001	
**Tb8 vs. Control**	2.167	1.704 to 2.629	****	<0.0001	
**Tb6 vs. Tb4**	0.5400	0.07730 to 1.003	*	0.0178	
**Tb6 vs. Tb2**	0.7400	0.2773 to 1.203	**	0.0013	
**Tb6 vs. Tb0 (HEC16)**	0.7233	0.2606 to 1.186	**	0.0016	
**Tb6 vs. Control**	1.100	0.6373 to 1.563	****	<0.0001	
**Tb4 vs. Tb2**	0.2000	−0.2627 to 0.6627	ns	0.7538	
**Tb4 vs. Tb0 (HEC16)**	0.1833	−0.2794 to 0.6460	ns	0.8166	
**Tb4 vs. Control**	0.5600	0.09730 to 1.023	*	0.0136	
**Tb2 vs. Tb0 (HEC16)**	−0.01667	−0.4794 to 0.4460	ns	>0.9999	
**Tb2 vs. Control**	0.3600	−0.1027 to 0.8227	ns	0.1809	
**Tb0 (HEC16) vs. Control**	0.3767	−0.08603 to 0.8394	ns	0.1486	

**Table A9:** Two-way ANOVA analysis and post-hoc Tuckey’s multiple comparison to evaluate the effect of time and oviposition substrates on T/C. *p* > 0.0123 = not significant (ns); *p* < 0.0021 **; *p* < 0.0001 = ****.

Ordinary Two-Way ANOVA					
**Alpha**	0.05				
**Source of Variation**	% of total variation	*p* value	*p* value summary		
**Interaction**	1.265	<0.0001	****		
**Substrate type**	97.92	<0.0001	****		
**Week**	0.3163	0.0020	**		
**ANOVA table**					
	SS	DF	MS	F (DFn. DFd)	*p* value
**Interaction**	0.9577	4	0.2394	F (4, 20) = 12.68	*p* < 0.0001
**Substrate type**	74.11	4	18.53	F (4, 20) = 981.3	*p* < 0.0001
**Week**	0.2394	1	0.2394	F (1, 20) = 12.68	*p* = 0.0020
**Residual**	0.3776	20	0.01888		
**Tukey’s multiple comparisons test**					
**Number of families**		1			
**Number of comparisons per family**		45			
**Alpha**		0.05			
		Mean Diff.	95% CI of diff.	Summary	Adjusted *p* Value
**Masonite:week1 vs. Masonite:week2**		0.000	−0.3973 to 0.3973	ns	>0.9999
**Masonite:week1 vs. HEC16:week1**		−0.1933	−0.5906 to 0.2039	ns	0.7709
**Masonite:week1 vs. HEC16:week2**		−0.1933	−0.5906 to 0.2039	ns	0.7709
**Masonite:week1 vs. HEC16/S6:week1**		−0.2167	−0.6139 to 0.1806	ns	0.6504
**Masonite:week1 vs. HEC16/S6:week2**		−1.110	−1.507 to −0.7127	****	<0.0001
**Masonite:week1 vs. HEC16/Tb8:week1**		−4.110	−4.507 to −3.713	****	<0.0001
**Masonite:week1 vs. HEC16/Tb8:week2**		−4.110	−4.507 to −3.713	****	<0.0001
**Masonite:week1 vs. Control:week1**		−0.04333	−0.4406 to 0.3539	ns	>0.9999
**Masonite:week1 vs. Control:week2**		−0.04333	−0.4406 to 0.3539	ns	>0.9999
**Masonite:week2 vs. HEC16:week1**		−0.1933	−0.5906 to 0.2039	ns	0.7709
**Masonite:week2 vs. HEC16:week2**		−0.1933	−0.5906 to 0.2039	ns	0.7709
**Masonite:week2 vs. HEC16/S6:week1**		−0.2167	−0.6139 to 0.1806	ns	0.6504
**Masonite:week2 vs. HEC16/S6:week2**		−1.110	−1.507 to −0.7127	****	<0.0001
**Masonite:week2 vs. HEC16/Tb8:week1**		−4.110	−4.507 to −3.713	****	<0.0001
**Masonite:week2 vs. HEC16/Tb8:week2**		−4.110	−4.507 to −3.713	****	<0.0001
**Masonite:week2 vs. Control:week1**		−0.04333	−0.4406 to 0.3539	ns	>0.9999
**Masonite:week2 vs. Control:week2**		−0.04333	−0.4406 to 0.3539	ns	>0.9999
**HEC16:week1 vs. HEC16:week2**		0.000	−0.3973 to 0.3973	ns	>0.9999
**HEC16:week1 vs. HEC16/S6:week1**		−0.02333	−0.4206 to 0.3739	ns	>0.9999
**HEC16:week1 vs. HEC16/S6:week2**		−0.9167	−1.314 to −0.5194	****	<0.0001
**HEC16:week1 vs. HEC16/Tb8:week1**		−3.917	−4.314 to −3.519	****	<0.0001
**HEC16:week1 vs. HEC16/Tb8:week2**		−3.917	−4.314 to −3.519	****	<0.0001
**HEC16:week1 vs. Control:week1**		0.1500	−0.2473 to 0.5473	ns	0.9325
**HEC16:week1 vs. Control:week2**		0.1500	−0.2473 to 0.5473	ns	0.9325
**HEC16:week2 vs. HEC16/S6:week1**		−0.02333	−0.4206 to 0.3739	ns	>0.9999
**HEC16:week2 vs. HEC16/S6:week2**		−0.9167	−1.314 to −0.5194	****	<0.0001
**HEC16:week2 vs. HEC16/Tb8:week1**		−3.917	−4.314 to −3.519	****	<0.0001
**HEC16:week2 vs. HEC16/Tb8:week2**		−3.917	−4.314 to −3.519	****	<0.0001
**HEC16:week2 vs. Control:week1**		0.1500	−0.2473 to 0.5473	ns	0.9325
**HEC16:week2 vs. Control:week2**		0.1500	−0.2473 to 0.5473	ns	0.9325
**HEC16/S6:week1 vs. HEC16/S6:week2**		−0.8933	−1.291 to −0.4961	****	<0.0001
**HEC16/S6:week1 vs. HEC16/Tb8:week1**		−3.893	−4.291 to −3.496	****	<0.0001
**HEC16/S6:week1 vs. HEC16/Tb8:week2**		−3.893	−4.291 to −3.496	****	<0.0001
**HEC16/S6:week1 vs. Control:week1**		0.1733	−0.2239 to 0.5706	ns	0.8583
**HEC16/S6:week1 vs. Control:week2**		0.1733	−0.2239 to 0.5706	ns	0.8583
**HEC16/S6:week2 vs. HEC16/Tb8:week1**		−3.000	−3.397 to −2.603	****	<0.0001
**HEC16/S6:week2 vs. HEC16/Tb8:week2**		−3.000	−3.397 to −2.603	****	<0.0001
**HEC16/S6:week2 vs. Control:week1**		1.067	0.6694 to 1.464	****	<0.0001
**HEC16/S6:week2 vs. Control:week2**		1.067	0.6694 to 1.464	****	<0.0001
**HEC16/Tb8:week1 vs. HEC16/Tb8:week2**		0.000	−0.3973 to 0.3973	ns	>0.9999
**HEC16/Tb8:week1 vs. Control:week1**		4.067	3.669 to 4.464	****	<0.0001
**HEC16/Tb8:week1 vs. Control:week2**		4.067	3.669 to 4.464	****	<0.0001
**HEC16/Tb8:week2 vs. Control:week1**		4.067	3.669 to 4.464	****	<0.0001
**HEC16/Tb8:week2 vs. Control:week2**		4.067	3.669 to 4.464	****	<0.0001
**Control:week1 vs. Control:week2**		0.000	−0.3973 to 0.3973	ns	>0.9999

**Table A10:** Two-way ordinary ANOVA analysis to evaluate the effect of time and substrate on T/C in field oviposition assay and Tuckey’s multiple comparison analysis. *p* > 0.0123 = not significant (ns); *p* < 0.0001 = ****.

Ordinary Two-Way ANOVA					
**Alpha**	0.05				
**Source of Variation**	% of total variation	*p* value	*p* value summary		
**Interaction**	13.35	<0.0001	****		
**Substrate**	7.908	<0.0001	****		
**Week**	78.48	<0.0001	****		
**ANOVA table**	SS	DF	MS	F (DFn. DFd)	*p* value
**Interaction**	10.38	2	5.192	F (2, 12) = 309.2	*p* < 0.0001
**Substrate**	6.150	1	6.150	F (1, 12) = 366.2	*p* < 0.0001
**Week**	61.04	2	30.52	F (2, 12) = 1817	*p* < 0.0001
**Residual**	0.2015	12	0.01679		
**Tukey’s multiple comparisons test**					
**Number of families**		1			
**Number of comparisons per family**		15			
**Alpha**		0.05			
		Mean Diff.	95% CI of diff.	Summary	Adjusted *p* Value
**0–15 days: HEC16 vs. 0–15 days: HEC16/S6/TB8**		−0.2809	−0.6363 to 0.07451	ns	0.1569
**0–15 days: HEC16 vs. 0–15 days: Control**		2.548	2.193 to 2.904	****	<0.0001
**0–15 days: HEC16 vs. 15–30 days: HEC16**		−0.1927	−0.5481 to 0.1627	ns	0.4884
**0–15 days: HEC16 vs. 15–30 days: HEC16/S6/TB8**		−3.595	−3.951 to −3.240	****	<0.0001
**0–15 days: HEC16 vs. 15–30 days: Control**		2.548	2.193 to 2.904	****	<0.0001
**0–15 days: HEC16/S6/TB8 vs. 0–15 days: Control**		2.829	2.474 to 3.185	****	<0.0001
**0–15 days: HEC16/S6/TB8 vs. 15–30 days: HEC16**		0.08818	−0.2672 to 0.4436	ns	0.9552
**0–15 days: HEC16/S6/TB8 vs. 15–30 days: HEC16/S6/TB8**		−3.315	−3.670 to −2.959	****	<0.0001
**0–15 days: HEC16/S6/TB8 vs. 15–30 days: Control**		2.829	2.474 to 3.185	****	<0.0001
**0–15 days: Control vs. 15–30 days: HEC16**		−2.741	−3.097 to −2.386	****	<0.0001
**0–15 days: Control vs. 15–30 days: HEC16/S6/TB8**		−6.144	−6.499 to −5.789	****	<0.0001
**0–15 days: Control vs. 15–30 days: Control**		0.000	−0.3554 to 0.3554	ns	>0.9999
**15–30 days: HEC16 vs. 15–30 days: HEC16/S6/TB8**		−3.403	−3.758 to −3.047	****	<0.0001
**15–30 days: HEC16 vs. 15–30 days: Control**		2.741	2.386 to 3.097	****	<0.0001
**15–30 days: HEC16/S6/TB8 vs. 15–30 days: Control**		6.144	5.789 to 6.499	****	<0.0001
